# Inhibition of Phosphodiesterase 5 Promotes the Aromatase-Mediated Estrogen Biosynthesis in Osteoblastic Cells by Activation of cGMP/PKG/SHP2 Pathway

**DOI:** 10.3389/fendo.2021.636784

**Published:** 2021-03-12

**Authors:** Wisanee Wisanwattana, Kanjana Wongkrajang, Dong-yi Cao, Xiao-ke Shi, Zhong-hui Zhang, Zong-yuan Zhou, Fu Li, Qing-gang Mei, Chun Wang, Apichart Suksamrarn, Guo-lin Zhang, Fei Wang

**Affiliations:** ^1^ Center for Natural Products Research, Chengdu Institute of Biology, Chinese Academy of Sciences, Chengdu, China; ^2^ University of Chinese Academy of Sciences, Beijing, China; ^3^ Department of Chemistry, Faculty of Science and Technology, Pibulsongkram Rajabhat University, Phitsanulok, Thailand; ^4^ College of Chemical Engineering, Sichuan University, Chengdu, China; ^5^ Department of Chemistry and Center of Excellent for Innovation in Chemistry, Faculty of Science, Ramkhamhaeng University, Bangkok, Thailand

**Keywords:** icariin analogs, aromatase, osteoblast, PDE5, estrogen biosynthesis

## Abstract

Mechanical stimulation induces bone growth and remodeling by the secondary messenger, cyclic guanosine 3’, 5’-monophosphate (cGMP), in osteoblasts. However, the role of cGMP in the regulation of estrogen biosynthesis, whose deficiency is a major cause of osteoporosis, remains unclear. Here, we found that the prenylated flavonoids, 3-*O*-methoxymethyl-7-*O*-benzylicaritin (13), 7-*O*-benzylicaritin (14), and 4'-*O*-methyl-8-isopentylkaempferol (15), which were synthesized using icariin analogs, promoted estrogen biosynthesis in osteoblastic UMR106 cells, with calculated EC_50_ values of 1.53, 3.45, and 10.57 µM, respectively. 14 and 15 increased the expression level of the bone specific promoter I.4-driven aromatase, the only enzyme that catalyzes estrogen formation by using androgens as substrates, in osteoblastic cells. 14 inhibited phosphodiesterase 5 (PDE5), stimulated intracellular cGMP level and promoted osteoblast cell differentiation. Inhibition of cGMP dependent-protein kinase G (PKG) abolished the stimulatory effect of 14 on estrogen biosynthesis and osteoblast cell differentiation. Further, PKG activation by 14 stimulated the activity of SHP2 (Src homology 2 domain-containing tyrosine phosphatase 2), thereby activating Src and ERK (extracellular signal-regulated kinase) signaling and increasing ERK-dependent aromatase expression in osteoblasts. Our findings reveal a previously unknown role of cGMP in the regulation of estrogen biosynthesis in the bone. These results support the further development of 14 as a PKG-activating drug to mimic the anabolic effects of mechanical stimulation of bone in the treatment of osteoporosis.

## Introduction

Osteoporosis is a major global public health problem caused by the reduced estrogen level in postmenopausal women (1). In humans, aromatase cytochrome P450 (CYP19A1) catalyzes the formation of estrogens from C19 androgens (2). The aromatase expression at various sites is regulated by tissue-specific promoters through the alternative splicing mechanisms (3). In bone, class I cytokines such as TGF-β1, IL-1β, and TNF-α drive aromatase expression by the usage of promoter I.4 (4). Aromatase activity is a key factor in skeletal development and mineralization, and is crucial to estrogen production in the bone (5). Aromatase activity may decline with an increase in during aging, and the contribution of such decline to age-related bone loss is similar in magnitude to that of sex steroid deficiency in both women and men (6, 7). Therefore, agonists of aromatase expression or activity in the bone would be a new therapeutic means for preventing and treating osteoporosis.

Mechanical stimulation is a primary determinant of bone growth and remodeling, through generating the shear stress that stimulates osteoblasts and osteocytes and enhances their anabolic activity. In mechanically stimulated osteoblasts the NO/cGMP/PKG signaling pathway activates Erk-1/2 and a proliferative response through the recruitment of PKGII, Src, and SHP-1/2 into an integrin β3-containing mechanosome membrane complex (8). NO, which is increased by estrogen exposure, also mediates estrogen-stimulated human and rodent osteoblast proliferation and differentiation (9). Src kinase has also been found to regulate aromatase activity by directly phosphorylating aromatase or indirectly regulating aromatase expression through the MAPK pathway (10). In osteocyte, 17β-estradiol is found to prevent the bone loss by increasing its survival through NO/cGMP-mediated stimulation of Akt and Akt- and PKG-dependent phosphorylation of the pro-apoptotic Bcl-2 protein BAD (11). However, the role of cGMP in the regulation of estrogen biosynthesis in osteoblasts is still not well understood. An inhibitor of PDE5, which is responsible for cGMP degradation, has been found to increase aromatase expression and estrogen biosynthesis in human adipocytes and ovarian granulosa cells (12, 13). Thus, it will be of interest to investigate the crosstalk of cGMP signaling on aromatase expression in osteoblastic cells, which may provide new insights into the underlying mechanism of cGMP-mediated signaling in osteoblast proliferation and differentiation.

It is unclear whether natural medicinal plants exert their antiosteoporotic effects by modulating estrogen biosynthesis in the bone. Previously we found that icariin from *Epimedium brevicornum*, a widely used antiosteoporotic medicinal plant, promotes the production of estrogen in human ovarian granulosa cells and osteoblastic cells, with an underlying mechanism that remains unclear (14). Icariin and its analogs are found to be the inhibitors of PDE5 (15). We showed that the PDE5 inhibitors, sildenafil and icariin analogs, promote aromatase expression in human ovarian granulosa-like KGN cells by activating the cAMP/CREB pathway (13). Thus, further investigating the effect and mechanism of icariin analogs on aromatase regulation in bone tissue will be important for developing new therapeutic means to prevent and treat osteoporosis.

## Materials and Methods

### Chemicals and Reagent

The 18 icariin analogs were synthesized and identified as described previously (16, 17, **Figure S1**). The compounds were dissolved in DMSO (Sigma-Aldrich, Shanghai, China) and stored at -20°C. Testosterone was purchased from Sigma-Aldrich. The magnetic particle-based 17β-estradiol enzyme-linked immunosorbent assay (ELISA) kit was purchased from Bio-Ekon Biotechnology (Beijing, China). NSC-87877, KT5823, PD98059 and Rp-8-pCPT-cGMPS were obtained from Tocris Bioscience (MN, USA). Antibodies used in this study as follow: aromatase (1:1,000, ab64881, Abcam, Shanghai, China), Src (1:1,000, 11097-1-AP, Proteintech, Wuhan, China), shp2 (1:2,000, 20145-1-AP, Proteintech, Wuhan, China), phospho-Src-Tyr^418^ (1:1,000, 11091, SAB, Nanjing, China), phospho-Src-Tyr^529^ (1:1,000, 11153-1, SAB, Nanjing, China), ERK1/2 (1:1,000, 48504-1, SAB, Nanjing, China), phospho-ERK1/2 (1:1,000, 12082-1, SAB, Nanjing, China) and PDE5A (1:1,000, 37810, SAB, Nanjing, China).

### Cell Culture

The rat osteoblast-like cell line (UMR106), murine osteoblast-like cell line (MC3T3-E1), human embryonic kidney 293T (HEK293T) and 293A (HEK293A) cell lines were obtained from the Cell Bank of Chinese Academy of Sciences (Shanghai, China). UMR106, HEK293T, and HEK293A cells were maintained in DMEM/High glucose medium supplemented with 10% (v/v) fetal bovine serum and 1% (v/v) penicillin/streptomycin at 37°C in 5% CO_2_. MC3T3-E1 cells were maintained in α-MEM medium supplemented with 10% (v/v) fetal bovine serum and 1% (v/v) penicillin/streptomycin at 37°C in 5% CO_2_. Aromatase-overexpressing HEK293A cells as described before (18).

### Cell-Based Estrogen Biosynthesis Assay

The assay was conducted as described previously (19). The UMR106 cells or MC3T3-E1 cells were seeded overnight in 24-well plates. After that the medium was replaced with serum-free medium, and the cells were pretreated for 24 h with the test chemicals. Testosterone (10 nM) was then added to each well, the cells were incubated for an additional 48 h. The magnetic particle-based ELISA kit was used to quantify the 17β-estradiol in the culture medium according to the manufacturer’s instructions (Bio-Ekon Biotechnology). The results were normalized to the total cellular protein content, and expressed as percentages of the control. The BCA protein assay kit was used for protein determination (Bestbio, Shanghai, China).

### Western Blotting

Immunoblotting was performed as described (19). Cells cultured were harvested in RIPA buffer supplemented with a protease inhibitor cocktail (Sigma). Proteins lysate was loaded and separated on a sodium dodecyl sulfate-polyacrylamide gel electrophoresis. After that, the proteins were blotted onto nitrocellulose membranes and then incubated with each specific antibody, then enhanced chemiluminescence detection (Amersham Biosciences, Piscataway, NJ, USA).

### Real-Time Quantitative Reverse Transcription-PCR

The qRT-PCR analysis was performed as described (19). TRIzol reagent was used to isolated total cellular RNA according to the manufacturer’s protocol (Invitrogen, Carlsbad, CA, USA). SuperScript III Reverse Transcriptase (Invitrogen, Carlsbad, CA, USA) was used to reverse-transcribe total RNA (2 μg) with oligo dT15 primer. Equal amounts (1 μL) of cDNA were subjected qRT-PCR with the florescent dye SYBR Green I, according to the manufacturer’s protocol (TransGen Biotech, Beijing, China). The following primer pairs were used: *aromatase*, 5’- ATGTTTCTGGAAATGCTGAACCCGATGCATT -3’ (forward) and 5’- CTGTTTCAGATATTTTTCGCTGTTGCGCGG -3’ (reverse); aromatase *promoter I.4*, 5'–CACTGGTCAGCCCATCAA–3’ (forward) and 5’- ACGATGCTGGTGATGTTATAATGT–3’ (reverse); *GAPDH*,:5’-GGTCAGTGCCGGCCTCGTCTCATAGACA–3’ (forward) and 5’-GAGGGTGCAGCGAACTTTATTGA–3’(reverse); *Runt-related transcription factor 2* (*Runx2*), 5’–ATGCTGCATAGCCCGCATAAACAGCCGCAG–3’ (forward) and 5’- GTTCGCATCCGGCGCCTGCGGCACGCTCTG–3’ (reverse); *Osteocalcin* (*OCN*), 5’–ATGCGCACCCTGAGCCTGCTGACC–3’ (forward) and 5’–CACGGTGGTGCCATAAATGCGTTTA–3’ (reverse); *Osterix (Osx)*, 5’–ATGGCGAGCAGCCTGCTGGAAGAAGAAG–3’ (forward) and 5’–AATTTCCAGCAGGTTGCTCTGTTCCGG–3’ (reverse); *Alkaline phosphatase (ALP)*, 5’–ATGATTCTGCCGTTTCTGGTGCTGGC–3’ (forward) and 5’–AAACAGGGTGCGCAGCGGAAACAG–3’ (reverse); *Osteoprotegerin (OPG)*, 5’–CTGTGCGTGCCGTGCCCGGATTATAGCTA–3’ (forward) and 5’–CAGGCAGCTAATTTTCACGCTCTGCA–3’ (reverse); *Receptor activator of nuclear factor kappa-B ligand (RANKL)*, 5’–ATGCGCCGCGCGAACCGCGATTATG–3’ (forward) and 5’–ATCAATATCCTGCACTTTAAACGCG–3’ (reverse), and *β-actin*, 5’–ATGGATGATGATATTGCGGCGCTGG–3’ (forward) and 5’– AAAGCATTTGCGATGCACAATGCTCG–3’ (reverse). The thermal cycling conditions consisted of an initial denaturation step at 95 °C for 10 s, followed by 40 cycles of 95 °C for 60 s, 54 °C for 30 s, and 72 °C for 30 s. The relative quantity (n-Fold) of *aromatase*, *CYP19I.4*, *RUNX2*, *OCN*, *Osx*, *ALP*, and *OPG*/*RANKL* mRNA was calculated by the Δ(ΔCt) method using GAPDH as a reference amplified from the same sample.

### Measurement of Intracellular cGMP Level

UMR106 cells seeded in 6-well plates overnight were treated with **14** and sildenafil for the indicated time. The pre-cooled PBS buffer (120-150 μL) was added in 1×10^6^ cells to keep the cells suspended. The cells were lysed with the repeated freeze-thaw process. After centrifugation for 10 min at 1500 × g at 2-8°C, the supernatants were collected to carry out the assay. The cGMP concentration was determined with a commercial cGMP enzyme immunoassay kit (Elabscience, Wuhan, China). Thereafter, the results were measured with Thermo Scientific Verioskan Flash Multimode Reader at a wavelength of 450 nm ± 2 nm.

### Alkaline Phosphatase (ALP) Activity Assay

ALP activity was performed as described (20). UMR-106 cells were seeded in serum-free medium in a 24-well plate overnight and treated with the test compounds for 48 h. After that a kit using para-nitrophenyl phosphate as substrate was used to assay the cell lysate. The OD value was measured at 405 nm with Thermo Scientific Verioskan Flash Multimode Reader. The results were expressed as percentages of the control and normalized on a protein basis.

### SHP2 Activity Assay

SHP2 was immunoprecipitated and its activity is assayed as described previously (21). UMR106 cells were lysed in RIPA buffer that contained a complete protease inhibitor cocktail after treatment with **14** and SHP2 inhibitor (NSC-87877) for 30 min. The lysates were incubated on ice for 10 min and centrifuged at 20,000 × g for 15 min at 4°C. SHP2 antibody was incubated with cleared lysates overnight at 4°C with agitation, followed by the incubation with the Protein A/G agarose (Santa Cruz Biotech, TX, USA). The immunoprecipitates were resuspended gently in reaction buffer (100 μL) and transferred to a 96-well plate. The DiFMUP was used as the substrate of SHP2 to measure its activity with a plate reader (Thermo Scientific Varioskan^®^ Flash).

### Cellular Thermal Shift Assay

Cellular thermal shift assay was conducted as described previously (21). HEK293T cells were collected in PBS supplemented with protease inhibitor cocktail. The freeze-thawed cell lysates were centrifuged at 20,000 g for 20 min at 4°C, diluted with PBS and divided into two aliquots; one aliquot was treated with DMSO while the other was treated with **14** (100 μM). For temperature response experiments, 50 µL of lysate was transferred to PCR tubes and heated for 3 min to various temperatures. After that the cell lysates were centrifuged at 20,000 g for 20 min at 4°C to separate the soluble fractions from the precipitates. The supernatants were dissolved in loading buffer and analyzed by western blotting. The dose effect of **14** on the stability of PDE5A or vinculin was evaluated in the same manner.

### Measuring Recombinant Expressed PDE5 Activity

The activity of recombinant expressed PDE5 (Enzo Biochem, Madison, USA) was evaluated using the PDE-Glo™ Phosphodiesterase assay (Promega Corporation, Madison, WI, USA). Aliquots of PDE-Glo™ reaction buffer containing appropriate amounts of purified human recombinants PDE5A were added to a 96-well plate. After the addition of diluted compounds to each well, cGMP™ solution was added to initiate the reaction. After an appropriate incubation, Kinase-Glo^®^ reagent was pipetted into each well and 10 min later luminescence was measured using a plate reader (Thermo Scientific Varioskan^®^ Flash).

### Molecular Docking

The crystal structure of PDE5 [PDB code: 2H42] was obtained from the Protein Data Bank. During the process, all water molecules were removed, and hydrogen atoms were added to the protein molecule. Autodock 4 was used to predict the interactions between compounds and protein structure of PDE5 according to the binding energy with the default setting.

### Cell Viability Assay

UMR 106 cells were plated at 0.5 × 10^4^ cells/well in 96-well plates with 100 μL medium. The different concentrations of **13**, **14**, and **15** were used to treat the cultured cells for 24 h. After that the medium was added with 10 μL of the Alamar blue reagent and incubated for another 2–4 h with the measurement of the relative fluorescence intensity in each well.

### Statistical Analysis

Statistical analysis was analyzed by GraphPad Prism 6 (GraphPad, La Jolla, CA, USA). The results are expressed as mean ± standard error of the mean (S.E.M.) of three independent experiments with individual values. Data were compared by one-way ANOVA followed by Dunnett’s *post hoc* test. A p-value of less than 0.05 was considered to indicate a significant difference relative to the control.

## Results

### Effect of Icariin Analogs on Estrogen Biosynthesis

**Figure 1:**
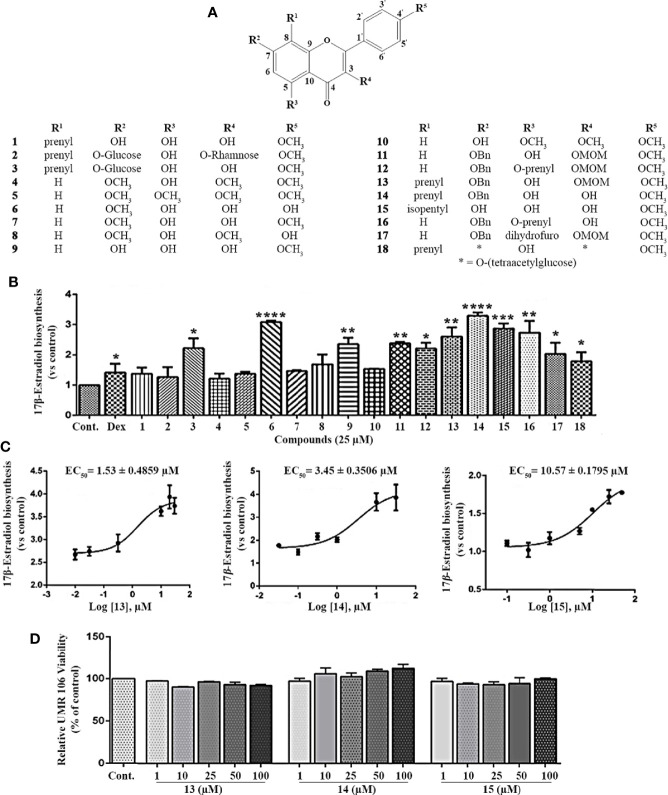
Effect of the icariin analogs on estrogen biosynthesis in osteoblast cells. **(A)** The chemical structure of icariin analogs. **(B)** UMR 106 cells seeded in 24-well plates were pretreated with the Dex (100 nM) and icariin analogs (25 μM) for 24 h. Subsequently, the cells were supplemented with testosterone (10 nM) for an additional 24 h and the 17β-estradiol (E_2_) concentration in the culture medium was quantified using a 17β-estradiol (E_2_) magnetic particle-based ELISA. **(C)** The concentration-response curve of compounds **13**, **14**, and **15** for the promotion of estrogen biosynthesis in UMR 106 cells. **(D)** Viability of UMR106 cells. UMR 106 cells grown in 96 well plates were pretreated with compounds **13**, **14**, and **15** (1-100 μM) for 24 h. Cell were then incubated with Alamar Blue reagent for an additional 4 h, and the fluorescence intensities were measured. Cont., DMSO-treated control; Dex, 100 nM dexamethasone. Error bars represent the standard deviation of the measurement. (*) p < 0.05, (**) p < 0.01, (***) p < 0.001 and (****) p < 0.0001 compared to the DMSO control.

To search for small molecules that modulate estrogen biosynthesis, we examined the effects of icariin analogs (16, 17, **Figure S1**) and their effects on estrogen biosynthesis in the rat osteoblast-like cell line, UMR106. The chemical structure of the icariin (**Figure 1A**, compound 2) and its analogs are presented in **Figure 1A**. As shown in **Figure 1B**, testosterone supplementation significantly increased 17β-estradiol production in UMR106 cells, which was further enhanced by dexamethasone treatment, thereby aligning with previous reports (22, 23). To examine the effect of icariin and its analogs on 17β-estradiol biosynthesis, UMR106 cells were incubated for 24 h with different concentrations of the test compounds followed by a further 24-h incubation with testosterone. Among the 18 compounds, 11 could increase the production of 17β-estradiol; these 9 compounds were the flavonoids icariside I (**3**), 7-*O*-methylkaempferol (**6**), kaempferide (**9**), 3-*O*-methoxymethyl-4^’^-*O*-methyl-7-*O*-benzylkaempferol (**11**), 3-*O*-methoxymethyl-4^’^-*O*-methyl-5-*O*-isopentenyl-7-*O*-benzylkaempferol (**12**), 3-*O*-methoxymethyl-7-*O*-benzylicaritin (**13**), 7-*O*-benzylicaritin (**14**), 4^’^-*O*-methyl-8-isopentylkaempferol (**15**), 4^’^-*O*-methyl-5-*O*-isopentenyl-7-*O*-benzylkaempferol (**16**), 4-benzyloxy-2,3,3-trimethyl-7-(4-methoxyphenyl)-8-methoxymethoxy-2,3-dihydrofuro[2,3-*f*]chromen-9-one (**17**) and 3-*O*-(2^’’’’^,3^’’’’^,4^’’’’^-tri-*O*-acetyl-α-L-rhamnopyranosyl)-7-*O*-(2^’’’^,3^’’’^,4^’’’^,6^’’’^-tetra-*O*-acetyl-β-D-glucopyranosyl)icaritin (**18**). Compounds **13**, **14**, and **15** promoted 17β-estradiol biosynthesis in a concentration-dependent manner, with EC_50_ values of 1.53, 3.45, and 10.57 µM, respectively (**Figure 1C**). They also had no effect on the viability of UMR106 cells (**Figure 1D**). These results indicate that the icariin analogs, such as compounds **13**, **14**, and **15**, could potently promote estrogen biosynthesis in rat osteoblast-like cells.

### Effect of Icariin Analogs on Aromatase Expression

**Figure 2:**
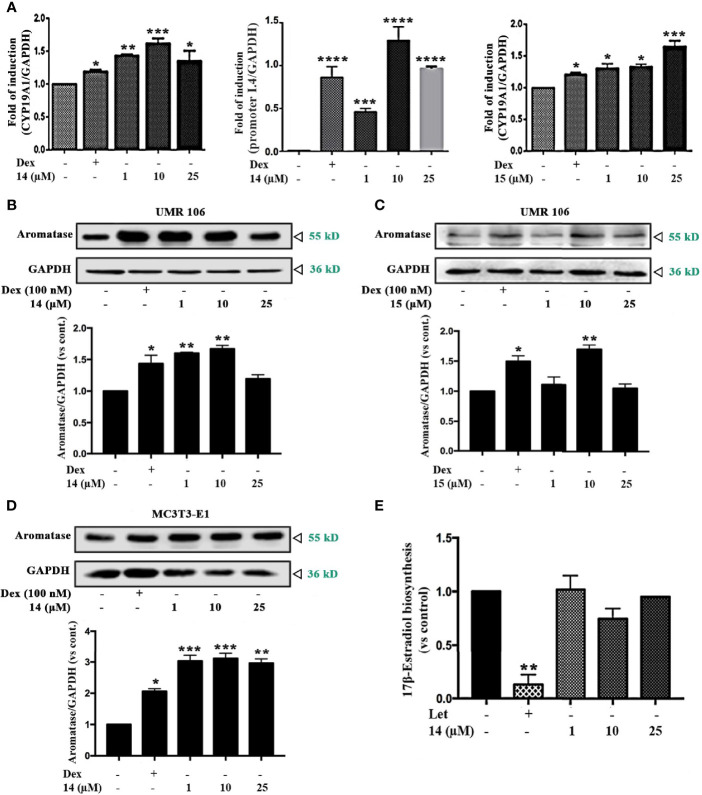
Effect of the icariin analogs on aromatase expression. **(A)** The mRNA expression of *aromatase* in UMR106 cells. UMR 106 cells were incubated with the indicated concentrations of **14** and **15** and Dex (100 nM) for 24 h. *Aromatase* mRNA was measured in total cellular RNA by real-time qPCR. The results are expressed as fold increase relative to levels in untreated cells. *GAPDH* was used as an internal control. **(B, C)** UMR106 cells were treated with the indicated concentrations of **14** and **15** for 24 h. The cell lysates were immunoblotted with aromatase and GAPDH antibodies. **(D)** MC3T3-E1 cells were treated with the indicated concentrations of **14** for 24 h. The cell lysates were immunoblotted with aromatase and GAPDH antibodies. **(E)** Let (10 µM) and 14 at the indicated concentrations treated aromatase-overexpressing HEK293A cells seeded in 24 well plates for 24 h, and then supplemented with tesoterone (10 nM) for a further 24. The culture medium was quantified using a 17β-estradiol ELISA. Cont., DMSO control; Dex, 100 nM dexamethasone; Let, 10 µM letrozole. (*) p < 0.05, (**) p < 0.01, (***) p < 0.001 and (****) p < 0.0001 compared to the control.

To determine whether compounds **14** and **15** promoted 17β-estradiol biosynthesis by affecting aromatase, we examined the mRNA and protein levels of aromatase in UMR106 cells treated with the selected compounds. Compounds **14** and **15** significantly increased aromatase transcript levels in a concentration-dependent manner (**Figure 2A**). **14** increased 58% of the aromatase mRNA levels at 10 μM while **15** increased 60% of the aromatase mRNA levels at 25 μM compared in the DMSO-treated control cells. **14** and **15** also significantly increased the bone specific aromatase promoter I.4 transcript in a concentration-dependent manner (**Figure 2A**, **Figure S2**). **14** and **15** also significantly increased aromatase protein expression in UMR106 and MC3T3-E1 cells in a concentration-dependent manner (**Figures 2B–D**). Furthermore, in aromatase-overexpressing HEK293A cells, letrozole, a specific inhibitor of aromatase enzymatic activity, significantly inhibited 17β-estradiol biosynthesis; however, **14** had no apparent effect on 17β-estradiol production compared to the DMSO-treated cells (**Figure 2E**), western blot also showed **14** had no effect on aromatase protein expression (**Figure S3**). These results excluding the probability that **14** directly modulates the enzymatic activity of aromatase protein. Actinomycin D, an RNA polymerase inhibitor, significantly suppressed **14**-induced aromatase mRNA transcription (**Figure S4**). These results indicate that **14** and **15** promoted estrogen biosynthesis by affecting aromatase at the transcriptional level.

### Inhibitory Effect of 14 on PDE5A Activity

**Figure 3:**
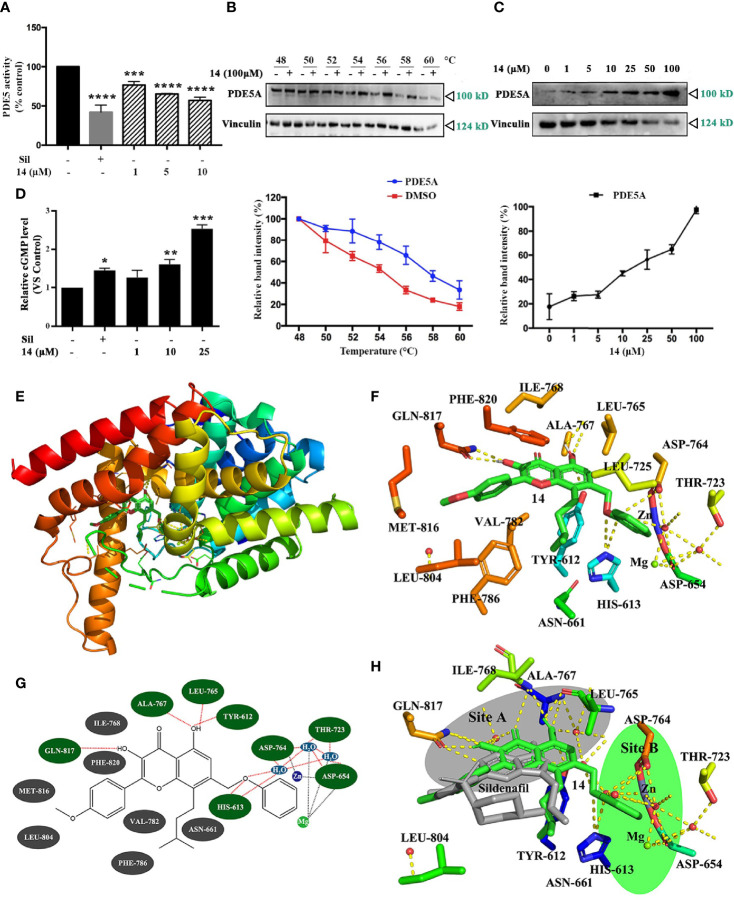
Inhibitory effect of **14** on PDE5. **(A)** The intracellular PDE5 level was detected. Basal PDE5 activity is normalized to a control. Cont., DMSO control; Sil, 10 µM sildenafil. (***) p <0.001 and (****) p <0.0001 compared to the DMSO control. **(B, C)** The cellular thermal shift assay was performed on HEK293T cells as described in Materials and Methods. The stabilization effect of **14** on PDE5A and vinculin at different temperatures **(B)** and different concentrations **(C)** was evaluated by western blot. Each experiment was repeated at least three times. **(D)** UMR106 cells were seeded in 6-well plates overnight were treated with the indicated concentration of 14 or 10 µM sildenafil for 24 h. The concentrations of intracellular cGMP were determined by ELISA as described in the materials and methods section. (*) p < 0.05, (**) p < 0.01, (***) p < 0.001 compared to the DMSO control. **(E)** Molecular docking model of PDE5 complexed with **14**. **(F)** Stereo view of the active site of the PDE5-**14** complex. **14** showed interaction with Gln817, Ala767, Leu765, Tyr612 and His613. The yellow dotted lines represent hydrogen bonds and coordination interaction, excluding the coordination bonds interaction with Asp654 and the water molecule are connected to the Zn^2+^and Mg^2+^. **(G)** Simplified structure showing interaction between **14** and amino acid residues at binding site. Hydrogen bond and hydrophobic interactions are colored in green and gray with red dotted line, respectively. **(H)** Comparison of sildenafil and **14** active sites in PDE5. Binding sites of sildenafil (colored gray) is site A and **14** (colored green) is site B.

Previously, the icariin analogs were found to be potent inhibitors of PDE5 (15); thus, we opted to use recombinant-expressed PDE5A to examine whether **14** inhibits PDE5 activity. As shown in **Figure 3A** and **Figure S5**, **14** significantly inhibited PDE5 activity in a concentration-dependent manner with the IC_50_ value of 9.914 μM, a finding similar to that obtained with the specific PDE5 inhibitor, sildenafil. **14** had no effect on the PDE5 expression in both UMR106 cells and MC3T3-E1 cells (**Figure S6**). Thereafter, we proceeded to perform a cellular thermal shift assay to examine whether **14** directly interacts with PDE5A in cells (24). Compared to the DMSO control, the presence of **14** markedly increased the accumulation of PDE5A in the soluble fraction at the temperatures examined (**Figure 3B**). We also tested the concentration-response of **14** on PDE5A stability at increased temperatures. An increase in **14** concentration resulted in a marked increase in PDE5A accumulation (**Figure 3C**). We then examined the effect of **14** on the intracellular cGMP level. As shown in **Figure 3D**, similar to sildenafil, **14** significantly stimulated the intracellular cGMP level in UMR106 cells. These findings suggest that **14** directly interacts with PDE5 and inhibits its activity in cells.

Computer docking analysis was conducted to assess the binding sites in PDE5. Based on our results, **14** fitted well within the active site of PDE5 (**Figure 3E**). The formation of hydrogen-bond (H-bond) and the hydrophobic interactions between **14** and PDE5 were evaluated. Two polar hydrogens in **14** are involved in its H-bonding with the amino acid Gln817, Ala767, Leu765, Tyr612 and His613, of PDE5 with a high glide energy of -11 kcal/mol^-1^. **14** also formed hydrophobic interactions with the residues Ile768, Phe820, Met816, Leu804, Val782, Phe786 and Asn661 (**Figures 3F, G**), which may contribute to its inhibition of PDE5. The binding sites of sildenafil (site A) and **14** (site B) were very close with binding sites near the zinc ions and magnesium ions. Sildenafil formed a hydrogen bond with the amino acid Gln817 (**Figure 3H**), thereby aligning with previous reports (25). These results suggest that **14** might be subjected to a nucleophilic attack in the PDE5 to inhibit its activity.

### Effect of 14 on Osteoblastic Cell Differentiation

**Figure 4:**
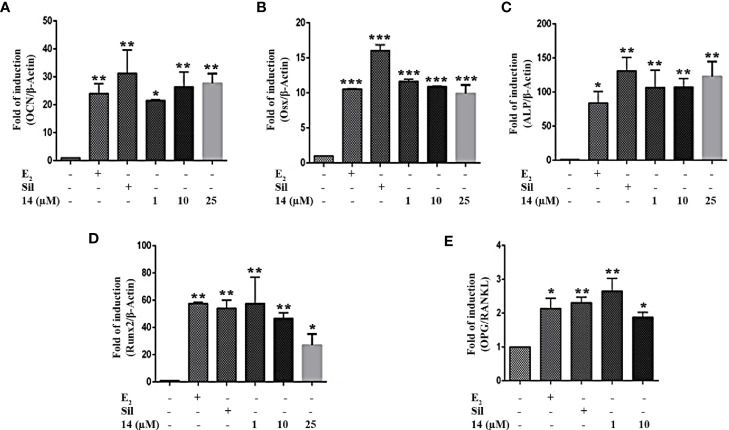
Effect of **14** on osteoblastic cell differentiation. The mRNA expression of **(A)** *OCN*, **(B)** *Osx*, **(C)** *ALP*, **(D)** *Runx2*, and **(E)** *OPG*/*RANKL* in UMR106 cells treated for 24 h. mRNA expression levels were quantified by qRT-PCR. The results are expressed as a fold increase relative to levels in untreated cells. *β*-*Actin* was used as the internal control. Quantitative results are depicted. Cont., DMSO-treated control; β-Actin control; OCN, osteocalcin; Osx, osterix; ALP, alkaline phosphatase; Runx2, Runt-related transcription factor 2; OPG, osteoprotegerin; RANKL, Receptor activator of nuclear factor-κB ligand; E_2_, 10 nM 17β-estradiol; Sil, 10 µM Sildenafil. (*) p < 0.05, (**) p < 0.01, (***) p < 0.001 and compared to the DMSO control.

To demonstrate that increased estrogen biosynthesis by **14** may promote osteoblastic cell differentiation, we examined the mRNA expression of osteoblastic cell differentiation markers in UMR106 cells. 17β-Estradiol significantly increased mRNA levels of *osteocalcin* (*OCN*), *osterix* (*Osx*), *alkaline phosphatase* (*ALP*), and *Runt-related transcription factor 2* (*Runx2*) (**Figures 4A–D**), aligning with the findings of previous reports (26, 27). Similar to sildenafil, **14** significantly increased the mRNA levels of *OCN*, *Osx*, *ALP*, and *Runx2* (**Figures 4A–D**). The bone formation/resorption balance can be observed from the ratio of *OPG*/*RANKL* expression, which is stimulated by 17β-estradiol (28). Similar to 17β-estradiol, both **14** and sildenafil increased the ratio of *OPG*/*RANKL* (**Figure 4E**). Compared with the control, calcium deposition was increased after treatment with **14** in a dose dependent manner (**Figure S7**). While **14** had no effect on BMP2 protein expression (**Figure S8**). These results indicate that **14** can promote osteoblast formation and differentiation.

### Effect of 14 on cGMP/PKG/Src/ERK Signaling

**Figure 5:**
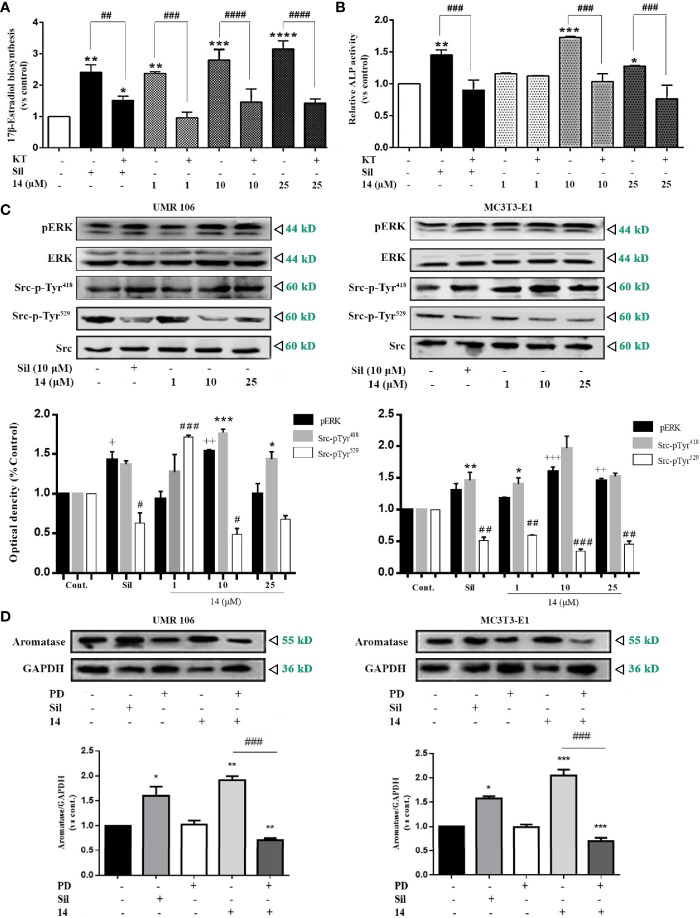
**14** activates PKG/Src/ERK pathway signaling. **(A)** UMR106 cells seeded in 24-well plates overnight were treated with compounds for 2 h. Subsequently, the cells were supplemented with testosterone (10 nM) for an additional 48 h. 17β-estradiol concentration in the culture medium was quantified with an ELISA (E2) detection kit. (*) p < 0.05, (**) p < 0.01, (***) p < 0.001 and (****) p < 0.0001 compared to the DMSO control; (##) p < 0.01, (###) p < 0.001 and (####) p < 0.0001 compared to KT5823 (10 μM)-treated cells **(B)** UMR106 cells seeded in 24-well plates overnight were treated with 14 and KT5823 (10 μM) for 2 h. Subsequently, the cells were supplemented with testosterone (10 nM) for an additional 48 h. ALP activity of the cell lysates was quantified with the ALP detection kit. (*) p < 0.05, (**) p < 0.01 and (***) p < 0.001 compared to the DMSO control; (###) p < 0.001 compared to KT-treated cells **(C)** UMR 106 and MC3T3-E1 cells were treated with different concentrations of 14 and sildenafil (10 μM) for 1 h. The cell lysates were immunoblotted with antibodies against phospho-ERK, ERK, phospho-Src-pTyr418, phospho-Src-pTyr529, and Src. (+) p < 0.05, (++) p < 0.01 and (+++) p < 0.001 compared to the p-ERK control; (*) p < 0.05, (**) p < 0.01, (***) p < 0.001 compared to the Src-pTyr418 control; (#) p < 0.05, (##) p < 0.01 and (###) p < 0.001 compared to the Src-pTyr529 control. **(D)** UMR 106 and MC3T3-E1 cells were treated with different concentrations of 14 and PD (10 μM) for 1 h. The cell lysates were immunoblotted with antibodies against aromatase. GAPDH was used as the internal control. Cont., DMSO-treated control; E2, 17β-estradiol (10 nM); Sil, sildenafil (10 μM); Rp-pCPT-cGMPS, ERK inhibitor (0.5 mM); KT, PKG inhibitor (KT5823, 10 μM); PD, ERK inhibitor (PD98059, 10 μM). Error bars represent the standard deviation of the measurement. (*) p < 0.05 (**) p < 0.01, (***) p < 0.001 compared to the DMSO control; (###) p < 0.001.

The stimulation of intracellular cGMP by the 14-induced PDE5 inhibition may activate PKG to increase aromatase expression. Therefore, we examined the role of PKG in the regulation of estrogen biosynthesis. PKG inhibition by the PKG inhibitor, KT5823 or Rp-8-pCPT-cGMPS, abolished the stimulatory effect of 14 and sildenafil on 17β-estradiol production (**Figure 5A** and **Figure S9**). PKG inhibition also abolished the stimulatory effect of **14** and sildenafil on ALP activity, a well-known marker of osteoblast differentiation (**Figure 5B**). These results suggest that PKG mediates the promotive effect of **14** on estrogen biosynthesis and differentiation in osteoblastic cells. As the cGMP-PKG signaling pathway activates Src and ERK in mechanically stimulated osteoblasts (8), we further examined the effect of **14** on Src and ERK in both UMR106 and MC3T3-E1 cells. Src activity is regulated by phosphorylation, where Tyr^529^ phosphorylation at the C-terminal retains Src in an inactive conformation. Dephosphorylation of Tyr^529^ is a key event in Src activation as it changes the protein to an active conformation and enables autophosphorylation of Tyr^418^ in the kinase domain activation loop (29). As shown in **Figure 5C**, **14** significantly promoted the Src phosphorylation on Tyr^529^ and decreased the phosphorylation on Tyr^418^, indicating Src activation. Similarly, **14** also promoted phosphorylation of ERK in both UMR106 and MC3T3-E1 cells. We treated the cells with a ERK inhibitor and found that it completely abolished the stimulatory effect of **14** on aromatase expression compared with **14** alone (**Figure 5D**), indicating that **14** enhances activation of ERK pathway signaling, thereby supporting the finding that inhibition of PKG abolished the stimulatory effect of **14** on estrogen biosynthesis (**Figure 5A**). These results suggest that **14** promotes osteoblast differentiation by activating the PKG/Src/ERK pathway.

### Effect of 14 on SHP2 Activation

**Figure 6:**
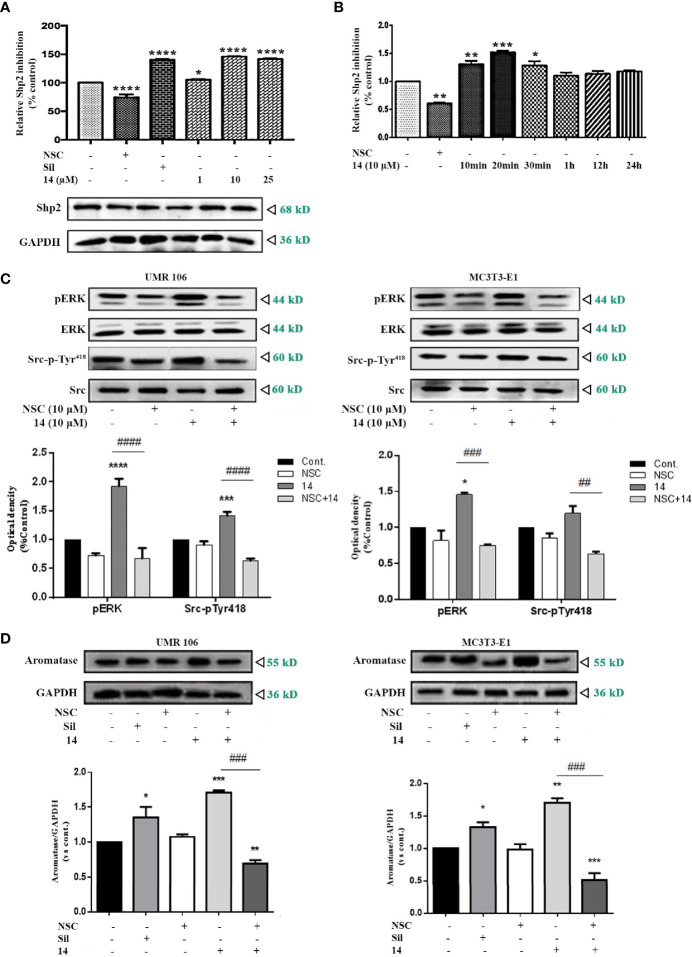
Effect of **14** on SHP2 activation. **(A)** UMR106 cells treated with compounds for 30 min were lysed and immunoprecipitated with the anti-SHP2 antibody. Thereafter, SHP2 activity was determined. GAPDH was used as the input. (*) p < 0.05 and (****) p < 0.0001 compared to the DMSO control. **(B)** Time-dependent effect of **14** on SHP2 activity. UMR106 cells treated with 10 μM NSC-87877 (30 min) and 10 μM **14** at the indicated time were lysed, immunoprecipitated with anti-SHP2 antibody, and SHP2 activity was determined. (*) p < 0.05, (**) p < 0.01 and (***) p < 0.001 compared to the DMSO control. **(C)** UMR106 and MC3T3-E1 cells were treated with compounds for 1 h. The cell lysates were immunoblotted with antibodies against phospho-ERK, ERK, phospho-Src-pTyr^418^, and Src. (*) p <0.05, (***) p < 0.001 and (****) p < 0.0001 compared to the DMSO control; (##) p < 0.01, (###) p < 0.001, (####) p < 0.0001 compared to compared to **14** alone treatment. **(D)** UMR106 and MC3T3-E1 cells were treated with **14** (10 µM) or sildenafil (10 µM) alone for 24 h, or pretreated with NSC-87877 for 1 h, and then **14** (10 µM) was added for additional 24 h. Cell lysates were immunoblotted with antibodies against aromatase. GAPDH was used as the internal control. (*) p < 0.05, (**) p < 0.01, (***) p < 0.001 compared to the DMSO control. (###) p < 0.001 compared to **14** alone treatment. Cont., DMSO-treated control; NSC, 10 μM NSC-87877; Sil, 10 μM sildenafil. Error bars represent the standard deviation of the measurement.

In osteoblasts, cGMP/PKG-induced Src activation is mediated by SHP-2 (8). Compared to the DMSO control, **14** significantly promoted SHP2 activity in the treated cells in a concentration-dependent manner, similar to sildenafil (**Figure 6A**). SHP2 activity was also stimulated by **14** in a time-dependent manner (**Figure 6B**). To further confirm the role of SHP2 in the **14**-enhanced Src/ERK pathway signaling, we examined the effect of **14** alone or in combination with the SHP2 inhibitor (NSC87877). Based on our findings, SHP2 inhibitor treatment completely eliminated the promotive effect of **14** on the phosphorylation of Src-pTy^418^ and phosphorylation of ERK (**Figure 6C**). Furthermore, SHP2 inhibitor treatment significantly decreased the stimulatory effect of **14** on aromatase expression in both UMR106 and MC3T3-E1 cells (**Figure 6D**). These results indicate that **14** stimulates aromatase expression by activating SHP2.

## Discussion

Icariin is the most abundant bioactive flavonoid contained in *E. brevicornum* (30, 31). Both icariin and *E. brevicornum* exhibit anti-osteoporotic effects *in vitro* and *in vivo* by stimulating osteoblast proliferation; these findings support the wide use of *E. brevicornum* in many Traditional Chinese Medicine formulas for the treat bone fracture and prevent osteoporosis (32–34). Of the 18 icariin analogs examined in the present study, 11 could increase estrogen biosynthesis in rat osteoblast-like UMR106 cells. This was consistent with a previous report where structure-activity relationship analysis suggested that prenylation at the C-8 and C-6 position was essential for promoting the differentiation of primary osteoblasts (35). In this study, we found that the prenyl group at the C-8 position was more potent than the prenyl group at the C-6 position for promoting estrogen biosynthesis. In the adipose tissue and bone, aromatase expression is stimulated primarily by class I cytokines through promoter I.4 (5). Consistently, we found that **14** potently promoted estrogen biosynthesis by increasing promoter I.4-driven aromatase mRNA and protein expression in osteoblastic UMR-106 and MC3T3-1 cells. Previously, we found that 2-phenylbenzo[b]furans might enhance estrogen biosynthesis *via* direct allosteric regulation of aromatase enzymatic activity (18, 19). However, in this study, we found that **14** had no effect on the catalytic activity of aromatase protein, excluding the probable role of **14** in the direct modulation of the catalytic activity of the aromatase protein. Local estrogen biosynthesis in bone plays a key role in bone homeostasis in postmenopausal women due to the loss of function of the ovary. As it is rarely reported that small chemical compounds could stimulate estrogen biosynthesis in osteoblasts, further developing **14** and its analogs as new antiosteoporotic therapeutics would be worthwhile.

Currently, several PDE5 inhibitors have been approved by the FDA for the treatment of erectile dysfunction and pulmonary arterial hypertension (36). PDE5 plays a key role in cGMP signaling; however, its role in estrogen biosynthesis in the bone has been rarely evaluated. In the present study, we found that the icariin analog, **14**, a validated PDE5 inhibitor with IC_50_ 9.914 ± 0.3325 μM, promoted estrogen biosynthesis in UMR 106 and MC3T3-E1 cells by enhancing aromatase expression in a similar manner to icariin (14). Earlier studies also revealed the importance of Gln817, Tyr612, Phe786, and Ala783 amino acid in PDE5-inhibitor interaction (25). Here, we found that both sildenafil and compound **14** could bind to these amino acids in PDE5. Additionally, **14** also differently binds to other amino acids in PDE5. Thus, further investigation is required to determine whether these amino acids also regulate PDE5 activity. PDE5 inhibitors, such as tadalafil and sildenafil, have been found to stimulate aromatase expression in human adipocytes (12), further supporting the role of PDE5 in the regulation of promoter I.4-driven aromatase expression. cGMP plays a key role in osteoblast differentiation by activating PKG (37). Icariin analogs, which inhibits PDE5 activity, was found to promote osteoblast differentiation and exhibit antiosteoporotic effect *in vivo* (32–34, 38), thereby aligning with our finding that **14** increased the expression of osteoblast differentiation markers. PDE5 inhibitors were also reported to exert beneficial effects on ovariectomy or glucocorticoid-induced osteoporosis in rats (39, 40). Furthermore, PDE5 inhibition was found to reduce bone mass by suppressing canonical Wnt signaling, indicating that long-term treatment with PDE5 inhibitors at high dosage may cause bone catabolism (41). Therefore, the role of PDE5 in the regulation of bone homeostasis should be further investigated to develop PDE5 inhibitors as new antiosteoporotic therapeutics. Recently it is reported that PDE5 inhibitors could enhance osteoblastic bone formation by targeting PDE5A and reverse osteopenia in ovariectomy mice by an osteogenic mechanism (42, 43). Therefore, our findings that PDE5 inhibitors promote estrogen biosynthesis provide new insights for the clinical benefits of PDE5 inhibitors in the treatment of osteoporosis. Moreover, the prenyl group contributes to higher osteogenic activity than do flavonoids possibly by modulating estrogen receptors (44, 45). Thus, whether **14** exhibits its osteogenic activity by a dual-functional modulator of PDE5 and estrogen receptor needs further investigation.

Mechanical stimulation, such as exercise, can strengthen bones and reduce the risk of fractures (46). Compressive forces generated by weight bearing and locomotion induce small bone deformations and increase interstitial fluid flow, thereby promoting anabolic responses in osteoblasts through different signal transduction pathways, including calcium channels, Raf-MEK-ERK cascade, and nitric oxide (NO) (47). Recently, the NO-cGMP-PKG pathway was reported to regulate osteoblast proliferation and differentiation through the formation of an Src-containing mechanosome (8). Consistent with this result, we found that **14** or sildenafil increased intracellular cGMP level and activated ERK and Src *via* PKG in osteoblasts. More interestingly, we found that PKG inhibition suppressed **14** or sildenafil-induced estrogen production in osteoblasts, which could be justified by our finding that **14** or sildenafil stimulated the activity of SHP2 that was directly phosphorylated by PKG and was required to activate Src (8). ERK has been implied to promote osteoblast differentiation by regulating ALP activity, integrin synthesis, focal adhesion kinase, Runx2 phosphorylation, and transcriptional activity (48–50). In this study, we found that the inhibition of ERK also attenuated the stimulatory effect of **14** on aromatase expression, providing new insight into mechanical stimulation in osteoblast differentiation. ERK could phosphorylate the glucocorticoid receptor and modulate its transcriptional activity (51). Therefore, it will be of interest to further investigate whether ERK modulates the glucocorticoid receptor or other transcriptional factors to stimulate promoter I.4-driven aromatase expression in osteoblasts. 17β-Estradiol can rapidly enhance aromatase enzymatic activity by increasing aromatase protein phosphorylation in breast cancer cell lines, which is mediated by Src (10). Thus, **14**-induced Src activation may also stimulate aromatase enzymatic activity to promote estrogen production, which should be further investigated. SHP-2 regulates cell survival and proliferation by the activation of the RAS-ERK signaling pathway (52). SHP2 is found to physically interact with the estrogen receptor, which is necessary for the synergistic and persistent activation of ERK by leptin and estrogen (53). cGMP/PKG-mediated SHP2 activation may also regulate the function of the estrogen receptor to exert its anabolic effect in osteoblasts. Therefore, a further investigation to determine whether mechanical stimulation also modulates local estrogen biosynthesis or estrogen receptor to exert its antiosteoporotic effect is warranted.

## Conclusions

**Figure 7:**
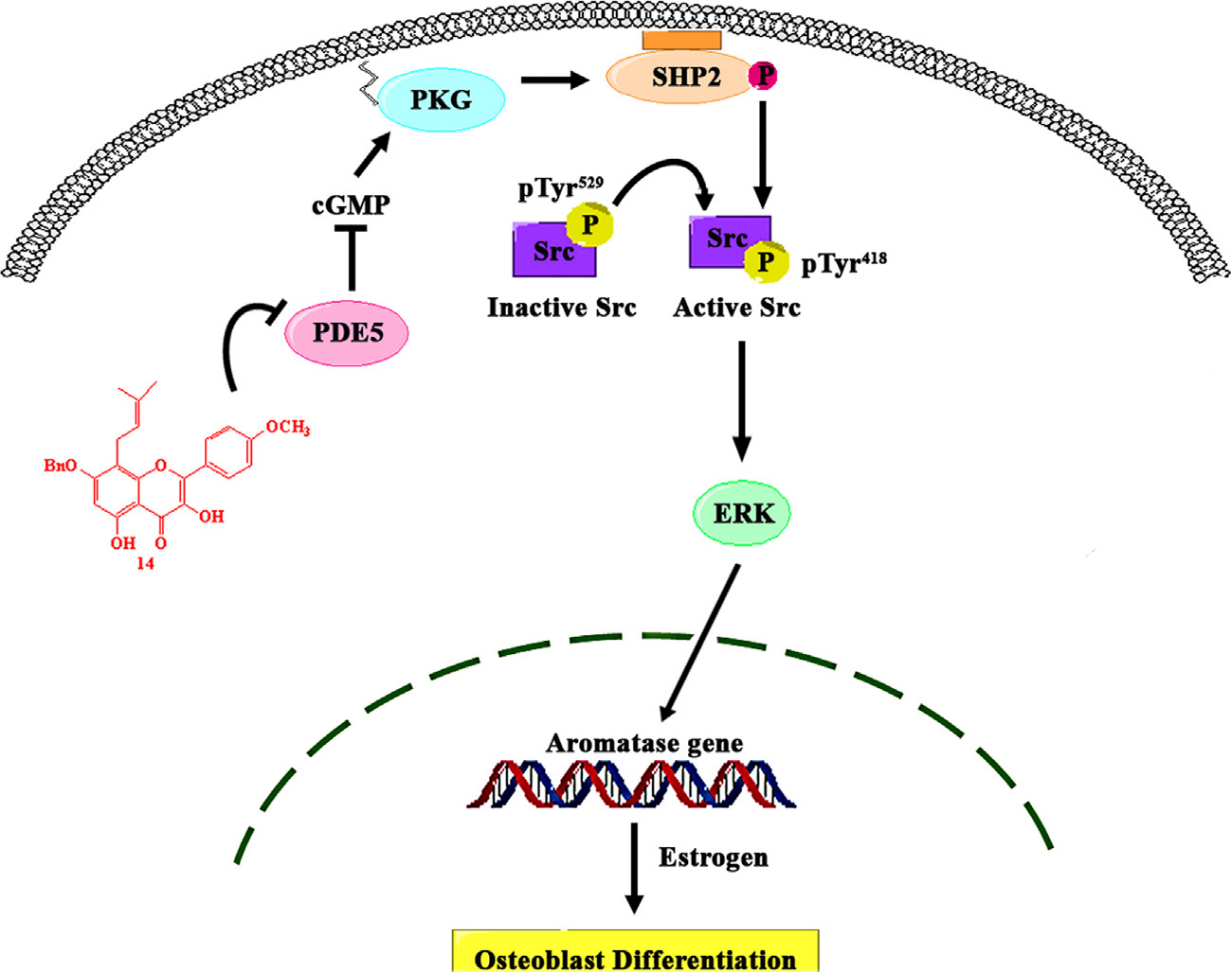
Proposed model of the role of PDE5 in the regulation of aromatase in osteoblasts. **14** inhibits the activity of PDE5, thereby stimulating the intracellular cGMP level, which causes PKG activation. PKG activation by **14** stimulated the activity of SHP2, which subsequently activated Src and ERK signaling and increased ERK-dependent gene expression, including that of aromatase, in osteoblasts.

In summary, we found that the prenylated flavonoid **14** promotes osteoblast differentiation by activating the cGMP/PKG/SHP2/Src/ERK cascade *via* PDE5 inhibition, thereby leading to the localized production of estrogen by stimulating aromatase expression (**Figure 7**). These data not only provide new insights into the role of estrogen biosynthesis in mechanical stimulation-induced osteoblast differentiation but also support the use of PDE5-inhibiting drugs to mimic the anabolic effects of mechanical bone stimulation in the treatment of osteoporosis.

## Data Availability Statement

The original contributions presented in the study are included in the article/**Supplementary Material**. Further inquiries can be directed to the corresponding authors.

## Author Contributions

WW, D-yC, and FW wrote the manuscript. WW, KW, D-yC, X-kS, and Z-yZ conducted the biological experiments. Z-hZ conducted the molecular docking analysis. FL, Q-gM, and CW synthesized the compounds. AS, G-lZ, and FW supervised the study, designed the experiments, and revised the manuscript. All authors contributed to the article and approved the submitted version.

## Funding

This work was supported by the National Natural Science Foundation of China (No. 21861142007, 21977092, 21550110193), Science & Technology Department of Sichuan Province (No. 2019YSF0106), CAS-TWAS President’s PhD Fellowship Program, Chinese Academy of Sciences President’s International Fellowship Initiative (No. 2016CTF092), Biological Resources Programme, Chinese Academy of Sciences (KFJ-BRP-008), and the National New Drug Innovation Major Project of China (2018ZX09711001-001-006). Support from The Thailand Research Fund (No. DBG6180030) is gratefully acknowledged.

## Conflict of Interest

The authors declare that the research was conducted in the absence of any commercial or financial relationships that could be construed as a potential conflict of interest.
