# Supplementary material for: Inhibition of Phosphodiesterase 5 Promotes the Aromatase-Mediated Estrogen Biosynthesis in Osteoblastic Cells by Activation of cGMP/PKG/SHP2 Pathway
Source: Front Endocrinol (Lausanne). 2021 Mar 12;12:636784. doi: 10.3389/fendo.2021.636784 (PMC7995890; doi:10.3389/fendo.2021.636784)
Supplement: Supplementary file 1 [file Image_1.pdf]

A

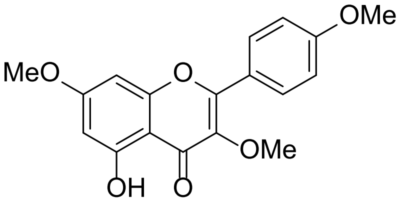

B

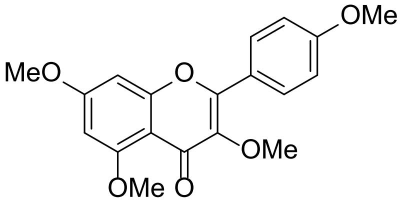

C

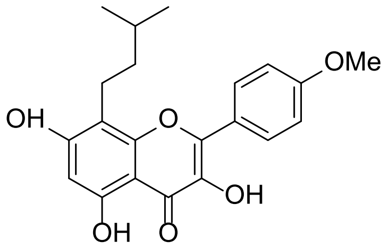

Figure S1. (A) Compound 3: ^{1}H NMR (400 MHz, DMSO-d_6): δ 12.63 (s, 1H), 8.06 (d, *J* = 9.0 Hz, 2H), 7.15 (d, *J* = 9.0 Hz, 2H), 6.77 (d, *J* = 2.1 Hz, 1H), 6.39 (d, *J* = 2.1 Hz, 1H), 3.86 (s, 6H), 3.81 (s, 3H). ^{13}C NMR (100 MHz, DMSO-d_6): δ 178.15, 165.22, 161.48, 160.96, 156.39, 155.58, 138.19, 130.09 (C-2′/6′), 122.11, 114.30 (C-3′/5′), 105.29, 97.86, 92.45, 59.81, 56.13, 55.50. ESI-HRMS *m/z*: 351.0826 $[M+Na]^+$ (calcd for $C_{18}H_{16}O_6Na$: 351.0839). **(B) Compound 4:** ^{1}H NMR (400 MHz, DMSO-d_6): δ 8.03 (d, *J* = 9.0 Hz, 2H), 7.12 (d, *J* = 9.0 Hz, 2H), 6.80 (d, *J* = 2.2 Hz, 1H), 6.49 (d, *J* = 2.2 Hz, 1H), 3.89 (s, 3H), 3.85 (s, 3H), 3.84 (s, 3H), 3.73 (s, 3H). ^{13}C NMR (150 MHz, DMSO-d_6): δ 172.03, 163.68, 160.88, 160.33, 158.18, 151.73, 140.23, 129.55 (C-2′/6′), 122.51, 114.14 (C-3′/5′), 108.50, 95.90, 92.98, 59.26, 56.11, 55.98, 55.39. ESI-HRMS *m/z*: 343.1180 $[M+H]^+$ (calcd for $C_{19}H_{19}O_6$: 343.1176). **(C) Compound 15:** ^{1}H NMR (400 MHz, CDCl3): δ 11.74 (s, 1H), 8.20 (d, J = 9.0 Hz, 2H), 7.05 (d, J = 9.0 Hz, 2H), 6.59 (s, 1H), 6.29 (s, 1H), 5.47 (s, 1H), 3.91 (s, 3H), 2.84 (t, J = 8.0 Hz, 2H), 1.73 – 1.68 (m, 1H), 1.53 – 1.48 (m, 2H), 1.02 (s, 3H), 1.01 (s, 3H). ESI-HRMS m/z: 393.1307 [M+Na] + (calcd for C21H22O6Na: 393.1309).

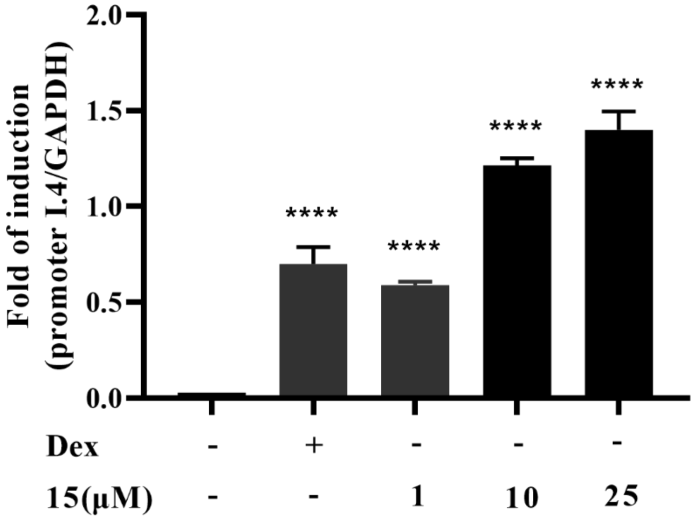

Figure S2. The mRNA expression of aromatase promoter I.4 in UMR106 cells. UMR 106 cells were incubated with the indicated concentrations of 15 and Dex (100 nM) for 24 h. Aromatase promoter I.4 mRNA was measured in total cellular RNA by real-time qPCR. The results are expressed as fold increase relative to levels in untreated cells. GAPDH was used as an internal control. (****) p <0.0001 compared to the DMSO control

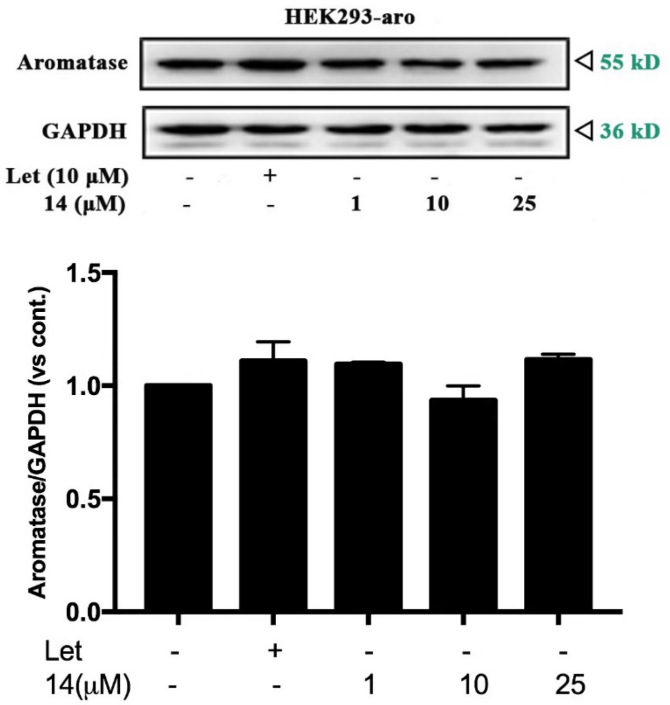

Figure S3. 14 had no effect on aromatase protein expression in aromatase-overexpressing HEK293A cells. Aromatase-overexpressing HEK293A cells were treated by Let (10 μM) and 14 at the indicated concentrations in 24-well plates for 24 h, and then supplemented with tesoterone (10 nM) for a further 24 h.The cell lysates were immunoblotted with anti-aromatase and anti-GAPDH antibodies. The quantitative results are depicted.

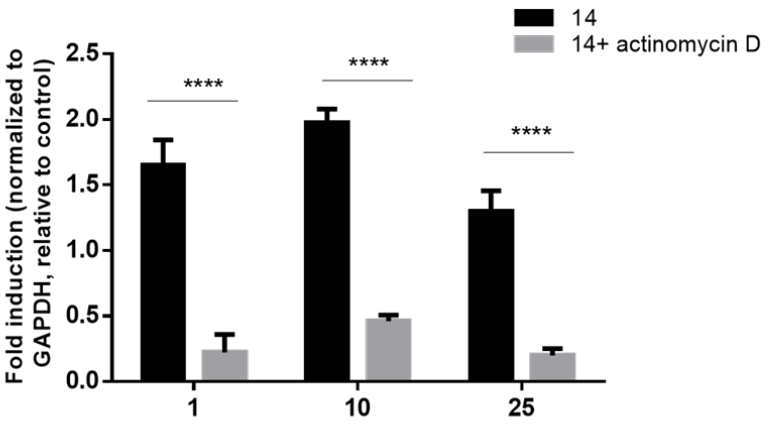

Figure S4. Effect of actinomycin D on 14-induced aromatase transcription**.** UMR 106 cells were incubated with the indicated concentration of 14 (1, 10, 25 μM) and 14 (1, 10, 25 μM) in combination actinomycin D (100 nM) for 24 h. The level of aromatase mRNA in total cellular RNA was measured by real-time qPCR. The results are expressed as fold increase relative to levels in untreated cells. GAPDH was used as an internal control.

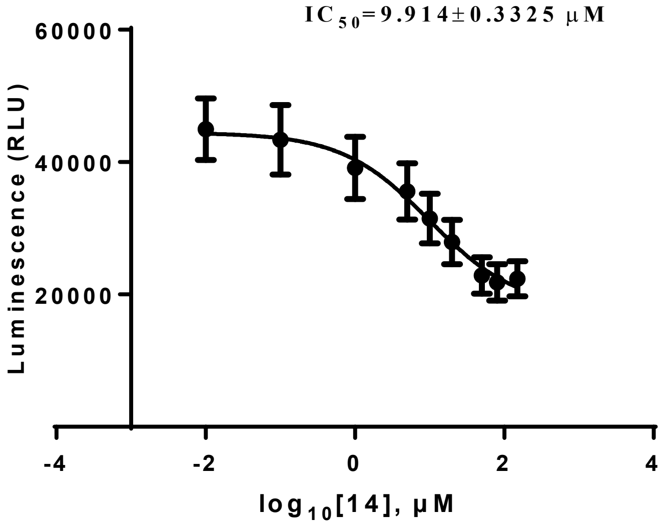

Figure S5. Effect of **14** on the inhibition of PDE5A activity. The concentration-response curve of **14** for the inhibition of recombinant expressed PDE5 protein was examined by PDE-glo™ phosphodiesterase assay.

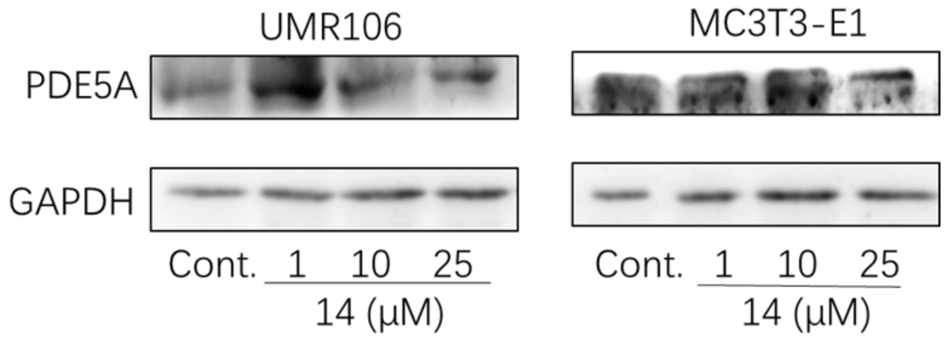

Figure S6. Effect of 14 on PDE5 expression. UMR106 and MC3T3-E1 cells were treated with the indicated concentrations of **14** for 24 h. The cell lysates were immunoblotted with PDE5A and GAPDH antibodies.

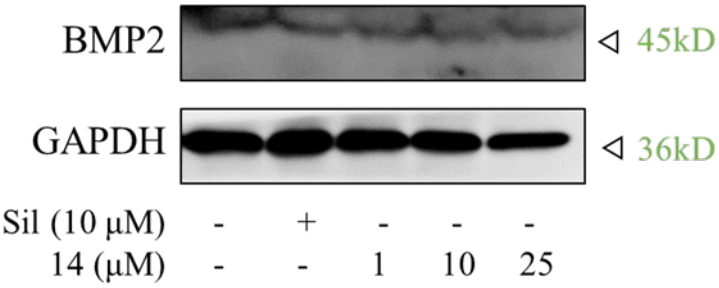

Figure S7. Effect of 14 on BMP2 expression. UMR106 cells were treated with the indicated concentrations of 14 for 24 h. The cell lysates were immunoblotted with BMP2 and GAPDH antibodies.

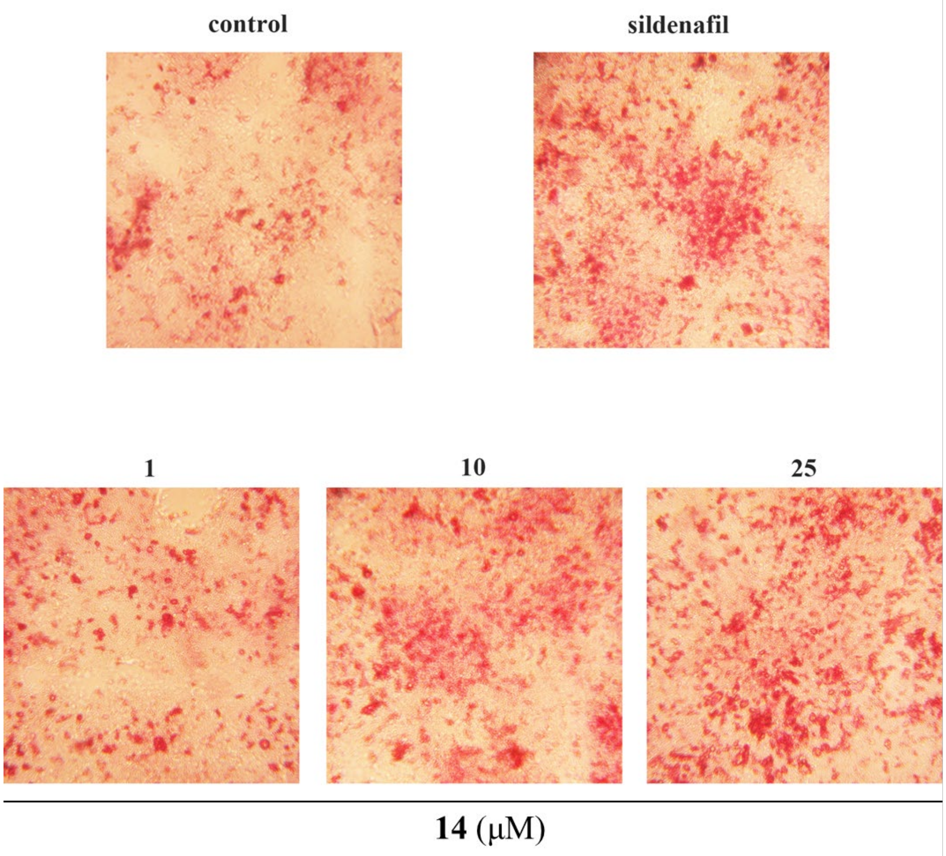

Figure S8. Compound 14 promoted the osteoblastic cell differentiation. UMR106 cells were seeded in 24-well plates and cultured overnight. Cells were treated with sildenafil (10 μM) and 14 (1, 10, or 25 μM)for 48 h, then replace the culture medium with differentiation medium (DMEM supplemented with 10 % FBS, 1 % penicillin/streptomycin, 50 μg/mL of ascorbic acid, 10 mM β-glycerophosphate, and 10 nM dexamethasone). The medium was refreshed every 3 days. On day 10, the cells were fixed with 4 % paraformaldehyde for 20 min, and then stained with 2 % Alizarin Red S for 10 min. Orange red parts represent the cell differentiation. Control, DMSO.

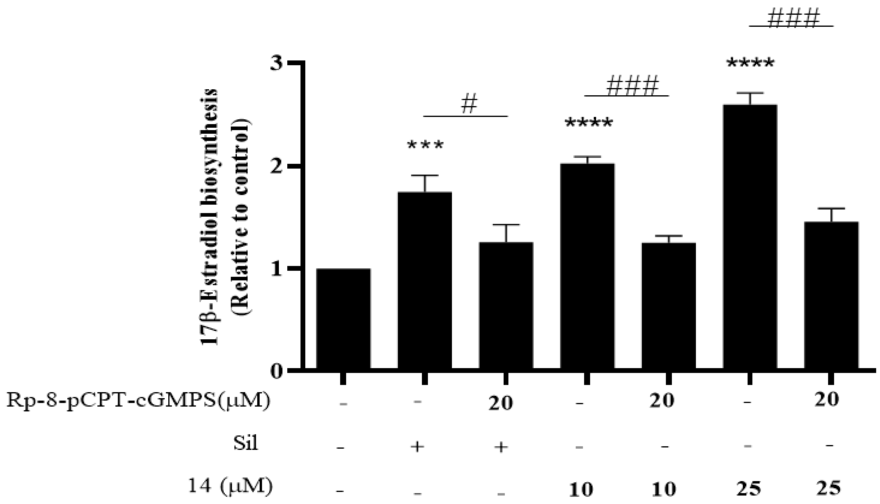

Figure S9. Effect of PKG inhibition on 14-induced estrogen biosynthesis. UMR106 cells were seeded in 24-well plates overnight. The medium was replaced to serum-free medium, and cells were treated with 14 and Rp-8-pCPT-cGMPS for 2 h. Testosterone was added to each wells, and cells were cultured for a further 48 h. 17β-estradiol concentration was quantified with an ELISA (E_2) detection kit. Sil, 10 μM sildenafil. (***) $p < 0.001$ and (****) $p < 0.0001$ compared to the DMSO control; (#) $p < 0.05$, (###) $p < 0.001$ compared to Rp-pCPT-cGMPS-treated cells.
